# Heavy metals in mining and agricultural ecotone: vertical distribution, ecological risks, and health implications

**DOI:** 10.1038/s41598-026-58692-8

**Published:** 2026-06-23

**Authors:** Yongjiang Zhang, Yijiao Guo, Zhengbo Xu, Fanjing Kong, Xixi Li, Yuwen Chen, Shuo Duan

**Affiliations:** 1 Department of Environment and Quality Test, Chongqing Chemical Industry Vocational College, Chongqing, 401220 China; 2 Chongqing Ecological Environment Monitoring Center, Chongqing, 401220 China; 3 Ecology and Environment Bureau of Dadukou District, Chongqing, 400000 China

**Keywords:** Heavy metal, Pollution source, Ecological risk, Health risk assessment, Vertical distribution, Ecology, Ecology, Environmental sciences, Risk factors

## Abstract

Heavy metal contamination in the mining–agriculture ecotones is influenced by lithogenic enrichment, industrial emissions, and agricultural inputs. Currently, knowledge of the vertical patterns and risk pathways in these environments is insufficient. In this study, paired soil profiles from mining and smelting areas of the manganese triangle (Xiushan, Chongqing, China) were analyzed to assess their physicochemical properties, metal concentrations, and ecological and health risks. The pH value was higher in the smelting soils (6.4–6.8) than in the mining soils (5.9–6.1). This similar trend was also observed in As and Pb, whereas contrary trend appeared in Zn and Cu. By contrast, Mn, Fe, Cr, Co, Sb, Se, and S concentrations remained stable across profiles, which indicated they had strong lithogenic controls. Pollution indices identified Sb (single-factor pollution index: < 9.15) and Cd (single-factor pollution index: 3.44–3.76) as dominant pollutants. The potential ecological risk index attributed 43.0% of the ecological risk in mining soils to Cd and 38.1% of the risk in smelting soils to Sb. Ecological risk classifications indicated moderate risks for smelting soils across depths, while mining soils exhibited greater variability with localized high-risk zones. In a human health risk assessment, the dominant contributors to carcinogenic and noncarcinogenic risks were ingestion (> 95%) and dermal contact (73%–80%), respectively, and inhalation had a negligible contribution. These results show mining- and smelting-derived metals have distinct vertical geochemical behaviors, which provides a mechanistic basis for targeted risk management in mining–agriculture ecotones.

**Supplementary Information:**

The online version contains supplementary material available at https://doi.org/10.1038/s41598-026-58692-8.

## Introduction

Heavy metal contamination is one of the most intractable global threats to soil quality, agricultural sustainability, and human health^1, 2, 3^. In contrast to organic pollutants that undergo degradation, heavy metals are inherently persistent, readily accumulate within soil–water–plant continua, and are biomagnified along food chains, thereby exerting long-term ecological and toxicological pressures^4, 5^. Hazardous elements such as cadmium (Cd), lead (Pb), arsenic (As), and mercury (Hg) are closely linked to carcinogenesis, nephrotoxicity, and neurodegenerative disorders^6, 7^. Physiologically essential elements, including iron (Fe), manganese (Mn), copper (Cu), and zinc (Zn), can also exert deleterious effects when present at supra-threshold concentrations^5, 8^. Intensification of mining and smelting and accelerated industrial discharges and application of agrochemicals have increasingly transformed farmland into a major sink of heavy metals via atmospheric deposition, wastewater irrigation, fertilizers, pesticides, and livestock manure^9, 10, 11^. In regions characterized by high population pressure and scarce arable land, understanding the vertical distributions, ecological risk pathways, and source contributions of soil heavy metals is imperative for ensuring food safety, maintaining ecosystem stability, and informing sustainable land restoration strategies.

Mining–agriculture ecotones are characterized by the dual influence of geological enrichment and anthropogenic disturbance, creating unique challenges for assessing heavy metal dynamics. Parent rocks in mining regions are naturally enriched in transition metals such as Fe, Mn, nickel (Ni), chromium (Cr), and cobalt (Co), leading to elevated background levels that complicate the distinction between lithogenic enrichment and anthropogenic inputs^12, 13^. Long-term mining and smelting activities continuously introduce high-risk elements, including Cd, Pb, As, and antimony (Sb), via tailings leaching, wastewater irrigation, and atmospheric deposition. In addition to accumulating in surface soils, these metals migrate vertically and redistribute over time^14, 15^. Agricultural practices further intensify the complexity of soil contamination. Fertilizers and manures introduce additional Cu, Zn, and selenium (Se, while pesticide residues facilitate the mobilization of Cd and Pb. Irrigation regimes, particularly continuous flooding in paddy fields and alternating wet and dry cycles, modify soil redox conditions and lead to dissolution of Fe and Mn oxides and subsequent release of adsorbed metals. These processes collectively enhance the mobility of As, Cd, Sb, and Se^16, 17, 18^. Contrasts between dryland and paddy field soils in structure, aeration, and microbial activity create strong heterogeneity in metal speciation and bioavailability, which complicates cross-land-use comparisons^19, 20^. Moreover, the persistence and non-degradability of heavy metals mean that soil concentrations reflect both historical legacies and recent inputs, while nonlinear regulatory effects of the pH, soil organic matter (SOM) content, and cation exchange capacity (CEC) add further uncertainty to mobility and risk prediction^21^ (Tchounwou et al.^22^). In such multi-source settings, overlapping signals from mining emissions, agricultural amendments, and parent material often obscure the precise sources for apportionment^23, 24^. Collectively, the challenges of research in mining–agriculture ecotones stem from the combined influence of multiple pollution sources, the inherent complexity of soil environments, and the multidimensional nature of risk assessment, which together complicate scientific interpretation and carry profound implications for pollution control and sustainable farmland management.

Although extensive studies have explored heavy metal pollution in mining regions, significant knowledge gaps and methodological limitations remain in elucidating the dynamics of mining–agriculture ecotones where extraction and smelting activities coexist^25, 26^. Early research has primarily focused on surface soils or single land-use types. Vertical soil profiles, which archive both legacy deposition and ongoing migration processes, have been neglected^27^. Conventional assessment methods, including the geo-accumulation index (*I*_geo_), Nemerow pollution index (PN), and related enrichment metrics, have provided valuable insight into contamination levels^10, 11, 28^ but fall short in revealing the underlying mechanisms of metal mobilization and redistribution. Advanced receptor models such as principal component analysis (PCA) and positive matrix factorization improve source apportionment^29^ but often neglect the nonlinear roles of soil properties, particularly pH, SOM, and the CEC, which strongly regulate metal mobility and bioavailability under contrasting redox conditions in paddy field and dryland soils^30, 31^. Consequently, while existing approaches have contributed substantially to risk characterization, they may under- or over-estimate real risks in complex ecotones, which underscores the need for integrative frameworks that combine profile-based monitoring with multivariate and nonlinear modeling to more accurately capture heavy metal dynamics^32^.

The “manganese triangle” of Xiushan County, Chongqing, China, is a typical mining–smelting–agriculture ecotone where farmland and industrial emissions converge to produce a complex mosaic of pollution sources and soil processes that remain poorly resolved. Within this context, the aim of the present study was to examine paired soil profiles from mining and smelting area to elucidate vertical and land-use differences in the physicochemical properties and multi-element distributions, assess contamination and ecological risks using multiple indices, and disentangle geochemical controls and source contributions using multivariate and nonlinear modeling. Additionally, human health implications were evaluated by partitioning exposure pathways. Through the integration of soil profile monitoring with advanced risk assessment and source apportionment approaches, this study aims to address the challenge of distinguishing natural and anthropogenic signals in complex ecotones, enhance the mechanistic understanding of heavy metal dynamics, and provide a scientific basis for farmland risk management and sustainable governance in mining-impacted regions.

## Materials and methods

### Sample collection

The sampling sites were located in Xiushan County, Chongqing, China, which is an area known as the manganese triangle (108°53′E, 28°31′N). The study area was located in a typical manganese mining region with agricultural land use and comprised two representative zones, hereafter referred to as the mining area and the smelting area. A total of 86 soil samples were collected, with 43 samples from each zone. Within each zone, soil samples were obtained from depths of 0–10, 10–20, 20–30, and 30–40 cm. Each sample weighed no less than 500 g and was stored in a clean, self-sealing bag for subsequent analysis.

### Determination of the soil pH, organic matter content, cation exchange capacity, and heavy metal concentrations

The soil pH was measured in a 1:2.5 (w/v) soil–water suspension using a calibrated pH electrode^33^. The SOM content was determined by dichromate oxidation and titration with ferrous ammonium sulfate^34^. The CEC was determined by saturation with 1 mol L^−1^ ammonium acetate at pH 7, followed by distillation and titration^35^.

Pseudo-total concentrations of As, Cd, Co, Cr, Cu, Fe, Mn, Ni, Pb, Sb, Se, S, and Zn were determined after microwave-assisted aqua regia digestion (HCl/HNO_3_ 3:1, v/v)^36, 37, 38^. Elemental concentrations were quantified by inductively coupled plasma mass spectrometry for trace metals and inductively coupled plasma atomic emission spectroscopy for Fe^39, 40^. A certified reference material (GBW07423), blank samples, and spiked samples were analyzed with every group of 10 samples. The recoveries were between 90%–110% and the relative standard deviations were < 10%.

### Pollution and ecological risk assessment indicators

Multiple classic indicators were used to comprehensively identify the pollution characteristics and potential ecological risks of heavy metals in soil from manganese mining areas. The indicators were the single-factor pollution index (*P*_*i*_), *I*_geo_, PN, and potential ecological risk index (PERI). These indicators reflect the enrichment degree and ecological risk level of heavy metals in soil from different perspectives and provide a scientific basis for subsequent environmental management and risk prevention and control.

#### *P*_*i*_

The *P*_*i*_ was first applied in environmental geochemistry to measure the enrichment degree of a single heavy metal element relative to the natural background of soil. This index is calculated as follows^9^ (Müller^41^):

1 $$P_{i} = \frac{{C_{i} }}{{B_{i} }},$$

where *C*_*i*_ is the measured mass concentration of element *i* in the soil (mg kg^−1^), and *B*_*i*_ is the regional soil background value of element *i* (mg kg^−1^). In this study, *B*_*i*_ was determined by combining background values of soil elements in China with the monitoring results for the study area. If *P*_*i*_ = 1, the element concentration is equivalent to that in the natural background, and if *P*_*i*_ > 1, the element concentration is enriched relative to that in the natural background. *P*_*i*_ results are generally classified as follows: 1 < *P*_*i*_ ≤ 2, mildly polluted; 2 < *P*_*i*_ ≤ 3, moderately polluted; and *P*_*i*_ > 3, severely polluted. This index can directly and clearly reflect the pollution status of a single element.

#### *I*_geo_

To further eliminate the influence of spatial differences in background values during natural soil formation on the results, Müller (1969) proposed using the *I*^42^_geo_. This index is calculated as follows (Loska et al.):

2 $$Igeo_{i} = \log_{2} \left( {\frac{{C_{i} }}{{1.5B_{i} }}} \right)$$

where 1.5 is an empirical correction coefficient used to compensate for the influence of lithological changes and background value fluctuations. The *I*_geo_ results are usually classified as follows: ≤ 0, not polluted; 0–1, not polluted to moderately polluted; 1–2, moderately polluted; 2–3, moderately to severely polluted; 3–4, severely polluted; 4–5, severely to very severely polluted; and > 5, very severely polluted^43^. Compared with *P*_*i*_, *I*_geo_ can better reflect the influence of the long-term geological background through logarithmic transformation and correction coefficients. This index is especially suitable when comparing the relative enrichment degrees of different regions or different metal elements. In this study, *I*_geo_ values were calculated for all detectable metal elements, including sulfur (S), to obtain a more complete pollution spectrum.

#### PN

Because it is difficult to fully evaluate the overall soil environmental quality using the degree of pollution with a single metal, the PN can be used to comprehensively consider the average *P*_*i*_ and the pollution level of the most serious element. The PN is calculated as follows^9^ (Müller 1969):

3 $$PN = \sqrt {\frac{{\left( {P_{i,\max } } \right)^{2} + \left( {P_{i,avg} } \right)^{2} }}{2}}$$

where *P*_*i*,max_ and *P*_*i*,avg_ are the maximum and average *P*_*i*_ values of all metals at the sampling point. The PN incorporates both the overall concentration of a metal and the influence of extreme values. This index can effectively characterize the comprehensive pollution status of regional soil. The results are generally classified as follows: PN ≤ 0.7, clean soil; 0.7–1.0, a warning level; 1–2, mildly polluted; 2–3, moderately polluted; and PN > 3, severely polluted^43^. The PN was used in this study to classify the level of pollution and evaluate spatial variation in soil pollution in the mining area.

#### PERI

Because it is difficult to evaluate the ecological toxicity caused by heavy metal pollution if only the metal concentrations are taken into account, Hakanson proposed using the PERI^44^. This index introduces an element toxicity coefficient that is based on the concentration and can provide a comprehensive assessment of potential risks. This method incorporates both the pollution degree and the ecological harmfulness of elements, which means it can identify the main risk-contributing metals and provide a quantitative basis for ecological restoration and management in mining areas. First, a single potential ecological risk coefficient (*E*_*r,i*_) is calculated as follows:

4 $$E_{r,i} = T_{i} \times \frac{{C_{i} }}{{B_{i} }}$$

where *T*_*i*_ is the toxicity response coefficient of element *i*. In this study, the following commonly used values in the literature were adopted: Cd, 30; As, 10; Pb, 5; Cu, 5; Ni, 5; Cr, 2; Zn, 1; Co, 5; Sb, 10; and Hg, 40. The comprehensive PERI is then calculated as the sum of the *E*_*r,i*_ values of the different elements^45^ as follows:

5 $${\mathrm{PERI}} = \mathop \sum \limits_{i} E_{r,i}$$

PERI results are classified as follows: < 150, low risk; 150–300, moderate risk; 300–600, relatively high risk; and ≥ 600 extremely high risk.

### Human health risk assessment

Three exposure pathways were considered: ingestion of soil particles, dermal contact with soil, and inhalation of resuspended soil dust. For each pathway, the average daily dose (ADD, mg kg^−1^ day^−1^) was estimated using the following equations^46^:

6 $${\mathrm{ADD}}_{ing} = \frac{C \times IR \times EF \times ED}{{BW \times AT}}$$

7 $${\mathrm{ADD}}_{dcrm} = \frac{C \times SA \times AF \times ABS \times EF \times ED}{{BW \times AT}}$$

8 $${\mathrm{ADD}}_{inh} = \frac{{C \times IR_{inh} \times EF \times ED}}{PERF \times BW \times AT}$$

where C is the concentration of metals in soil (mg kg^−1^); IR is the ingestion rate (mg day^−1^); SA is the surface area of skin exposed (cm^2^); AF is the soil adherence factor (mg cm^−2^); ABS is the dermal absorption factor (unitless); IR_inh_ is the inhalation rate (m^3^ day^−1^); PEF is the particle emission factor (m^3^ kg^−1^); EF is the exposure frequency (days year^−1^); ED is the exposure duration (years); BW is the body weight (kg); and AT is the averaging time (days).

The hazard quotient (HQ) for each metal and pathway was calculated as follows^47^:

9 $${\mathrm{HQ}} = \frac{ADD}{{Rfd}}$$

where Rfd is the reference dose (mg kg^−1^ day^−1^). The hazard index (HI) was obtained as the sum of HQs across all exposure pathways and metals.

The carcinogenic risk (CR) was estimated as follows^48^:

10 *CR = ADD × SF*

where SF is the slope factor [(mg kg^−1^ day^−1^) ^−1^]. The cumulative CR was the sum of risks across metals.

### Statistical analysis

Pearson correlation analysis was applied to evaluate the relationships between soil properties (pH, SOM, CEC, and depth) and the concentrations of potentially toxic elements (As, Cd, Co, Cr, Cu, Fe, Mn, Ni, Pb, Sb, Se, S, and Zn). Significance was determined at *P* < 0.05, 0.01, and 0.001. The results were visualized as heatmaps. Multivariate patterns were explored using PCA with standardized variables. The first two principal components (PC1 and PC2) were retained to illustrate the sample distributions and variation in loadings with site (mining vs. smelting) and depth (0–40 cm). Nonlinear effects of soil properties on metal concentrations were examined using generalized additive models with thin-plate regression splines implemented in the mgcv package (R v. 4.3.0), where the pH, SOM, and CEC were predictors, the metal concentrations were responses, and the site type was included as a categorical factor. Fitted relationships were visualized with 95% confidence intervals. Inter-group significance was assessed using the Wilcoxon test, and intra-group variation was assessed using *t*-tests (*P* < 0.05). All parameters were measured in triplicate. Correlation analysis was conducted with the Hmisc and corrplot packages. All figures were prepared in Adobe Illustrator 2022.

## Results

### Vertical distributions of soil properties and metal concentrations in mining and smelting areas

**Fig. 1:**
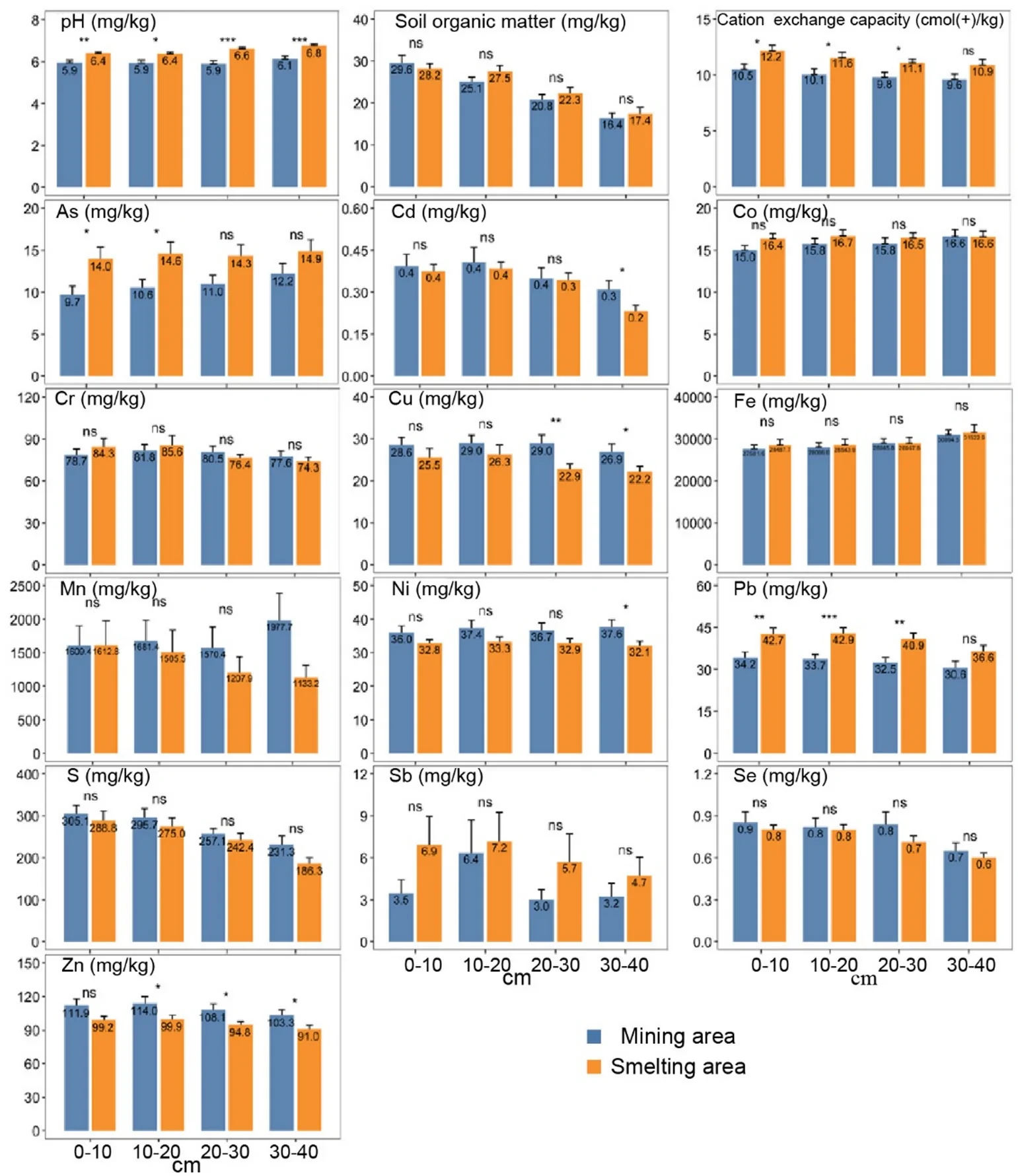
Vertical distribution of soil physicochemical properties (pH, SOM, CEC) and heavy metal concentrations (As, Cd, Co, Cr, Cu, Fe, Mn, Ni, Pb, Sb, Se, S, Zn) across four soil depths (0–10, 10–20, 20–30, 30–40 cm) in mining and smelting areas. Bars represent mean ± standard deviation. Statistical differences between areas are denoted as *p < 0.05, **p < 0.01, ***p < 0.001, and ns = not significant.

The pH, SOM content, CEC, and concentrations of As, Cd, Co, Cr, Cu, Fe, Mn, Ni, Pb, Sb, Se, S, and Zn exhibited clear vertical variations and site-specific differences between the mining and smelting areas (Fig. 1). Soil pH was consistently higher in the smelting area than in the mining area within the upper 30 cm of the soil, with values of 6.4 versus 6.9 at 0–10 cm (*P* < 0.05), 6.3 versus 6.8 at 10–20 cm (*P* < 0.001), and 6.5 versus 6.7 at 20–30 cm (*P* < 0.001). The soil pH values for the two areas converged at 6.8 in the 30–40 cm layer. The SOM content (17.4–29.6 mg kg^−1^) decreased slightly with depth but did not differ between the two areas. The CEC (10.5–12.2 cmol kg^−1^) was also stable across depths. The As concentration was markedly enriched in surface soils in the smelting area compared with the mining area (14.0 vs. 8.7 mg kg^−1^ at 0–10 cm, *P* < 0.05) but decreased with depth to 7.2–10.6 mg kg^−1^ and showed no differences between the two areas at soil depths below 10 cm. The Cd concentration remained very low (< 0.5 mg kg^−1^) in both areas at all depths. The difference in Cd concentrations between soil from the two areas was significant (*P* < 0.05) at a depth of 30–40 cm, with the smelting area soil containing 0.2 mg kg^−1^ Cd and the mining area soil containing 0.3 mg kg^−1^ Cd. The Cr (74–87 mg kg^−1^) and Co (~ 16 mg kg^−1^) concentrations exhibited no significant site- or depth-related changes.

The Cu concentrations in deeper soil layers in the smelting area were significantly lower than those in the mining areas. The values at 20–30 cm in the smelting and mining areas were 22.9 and 29.0 mg kg^−1^ (significantly different at *P* < 0.01), respectively. The values at 30–40 cm in the smelting and mining areas were 22.2 mg kg^−1^ and 28.5 mg kg^−1^ (significantly different at *P* < 0.05), respectively. The Ni concentrations showed a similar pattern, with a concentration of 32.1 mg kg ^−1^ in the smelting area at 30–40 cm compared with 37.6 mg kg^−1^ in the mining area (*P* < 0.05). By contrast, the concentrations of Mn (1133–1977 mg kg^−1^), Fe (~ 27,000 mg kg^−1^), S (186–305 mg kg^−1^), Sb (3–7 mg kg^−1^), and Se (0.6–0.9 mg kg^−1^) were relatively stable with depth and showed no site-related differences. The Pb concentration was highly enriched in surface soil in the smelting area, with concentrations of 42.7, 42.9, and 40.6 mg kg^−1^ at 0–10, 10–20, and 20–30 cm, respectively. These concentrations were significantly different from those at 0–10 cm (34.2 mg kg^−1^, *P* < 0.01), 10–20 cm (33.7 mg kg^−1^, *P* < 0.001), and 20–30 cm (32.5 mg kg^−1^, *P* < 0.01) in the mining area. At 30–40 cm, the Pb concentrations in both areas were similar (36.6 mg kg^−1^ in the smelting area and 30.8 mg kg^−1^ in the mining area). The Zn concentrations showed a similar trend, with concentrations of 99.2, 99.7, and 98.4 mg kg^−1^ in the smelting area at 0–10, 10–20, and 20–30 cm, respectively. By comparison, the concentrations in the mining area were significantly different (*P* < 0.05) at 111.9, 114.0, and 109.5 mg kg^−1^ in the 0–10, 10–20, and 20–30 cm layers, respectively. At 30–40 cm, the Zn concentrations in the smelting area (91.0 mg kg^−1^) and mining area (103.3 mg kg^−1^) were not significantly different. Overall, soils from the smelting area had lower pH values, higher concentrations of As, Pb, and Zn in the upper 30 cm, and lower concentrations of Cu and Ni compared with those from the mining area. Soils from the mining area had higher pH values and more stable vertical distributions of the metals than those from the smelting area. These findings indicate that the strongest effects of smelting processes occur within the topsoil layers.

### *P*_*i*_ values and dominant pollutants

**Fig. 2:**
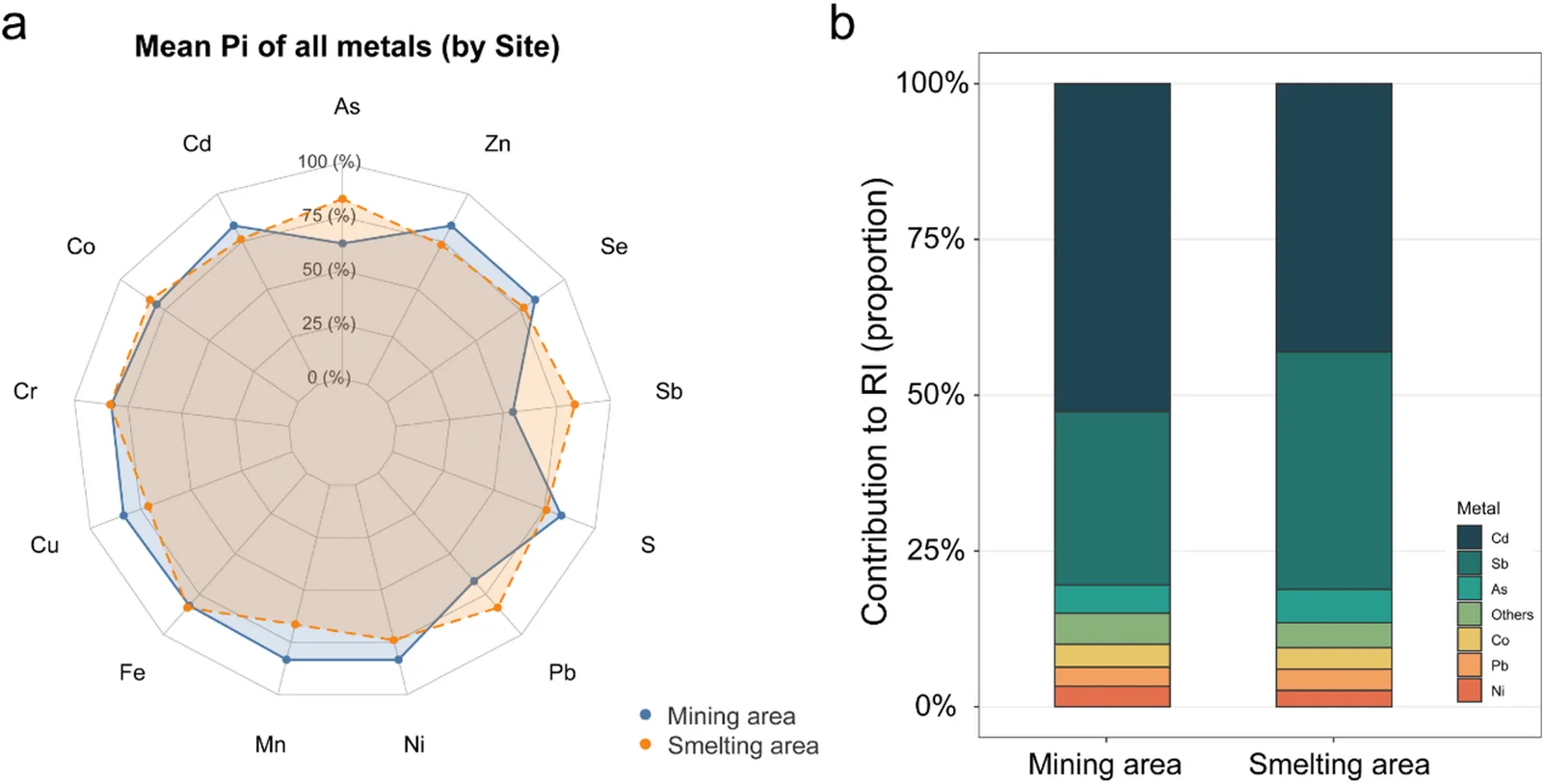
Mean single pollution index (*Pi*) values across the mining and smelting areas (**a**) and the contribution of individual factors to the total ecological risk (RI) at each site (**b**) are presented.

The mean *P*_*i*_ values of the metals exhibited clear differences between the mining and smelting areas (Fig. 2a). In the mining area, Sb had the highest *P*_*i*_ (5.98), followed by Cd (3.76), Mn (2.85), and Se (2.72). The lowest *P*_*i*_ values in the mining area were for S (0.78) and Fe (0.83). In the smelting area, the *P*_*i*_ for Sb reached 9.15, which was markedly higher than that in the mining area (5.98). The *P*_*i*_ values of As (1.29), Co (1.65), and Pb (1.63) were slightly higher in the smelting area than in the mining area, whereas those of Cd (3.44), Mn (2.27), Se (2.52), and Zn (1.44) were slightly lower in the smelting area than in the mining area. Overall, Sb was the dominant pollutant in both areas, with its enrichment particularly pronounced in the smelting area.

### Vertical distribution of PN

The distribution of PN across different soil depths in the mining and smelting areas revealed distinct trends (Fig. S1a). In the mining area, the majority of the soil samples across all depths (59.6%–73.2%) were severely polluted (PN > 3). There was a noticeable peak in the number of severely polluted samples at a depth of 0–10 cm. By comparison, fewer samples were classified as moderately (PN of 2–3) or mildly (PN of 1–2) polluted, especially in deeper soil layers (20–40 cm). In the smelting area, severe pollution dominated, with 78.3% of the samples severely polluted at 0–10 cm and 76.1% at 10–20 cm. Deeper soil layers (20–40 cm) showed a shift towards a higher proportion of moderately polluted soil (41.3%). The proportion of soils that was mildly polluted was minimal in both areas, particularly in the deeper soil layers. These results suggested that PN distribution in soil was influenced by both depth and site-specific factors, with the smelting area exhibiting more consistent dominance of severe pollution than the mining area.

### Vertical distribution of the PERI

Decomposition of the PERI revealed that Cd accounted for 52.6% and Sb for 27.9% of the total ecological risk in the mining area, whereas in the smelting area Sb contributed 38.1% and Cd 43.0% (Fig. 2b). The other metals had minor contributions to the total ecological risk in both areas. These findings demonstrate that Cd and Sb are the primary ecological risk drivers in the mining area and the smelting area, respectively.

Distribution of the PERI across different soil depths in the mining and smelting areas revealed distinct trends (Fig. S1b). In the mining area, most samples at a depth of 0–10 cm were classified as moderate risk (53.7% of the samples) or low risk (31.7% of the samples). Fewer samples were classified as relatively high risk (12.2% of the samples) or extremely high risk (2.4% of the samples). At a depth of 10–20 cm, 39% of the samples were classified as low risk, 39% as moderate risk, 9.8% as extremely high risk, and the remainder as relatively high risk. At depths of 20–30 and 30–40 cm, 51.2% and 56.1% of the samples were classified as moderate risk, respectively. The proportion of samples classified as low risk at both these depths was 36.6%. Few samples were classified as relatively high or extremely high risk at depths of 20–30 and 30–40 cm. In the smelting area, higher proportions of samples were classified as moderate risk across all depths compared with the mining area samples. This was especially apparent at a depth of 0–10 cm, where 65.2% of samples were classified as moderate risk. At a depth of 30–40 cm, 50% of the samples were classified as low risk. The only samples classified as extremely high risk in the smelting area occurred at a depth of 0–10 cm. The results showed a more consistent distribution of moderate and low risk across depths in the smelting area, while the mining area showed greater variability. In particular, higher proportions of samples classified as extremely high and relatively high risk were observed in surface soils in the mining area compared with the smelting area.

### Vertical distribution of *I*_geo_

**Fig. 3:**
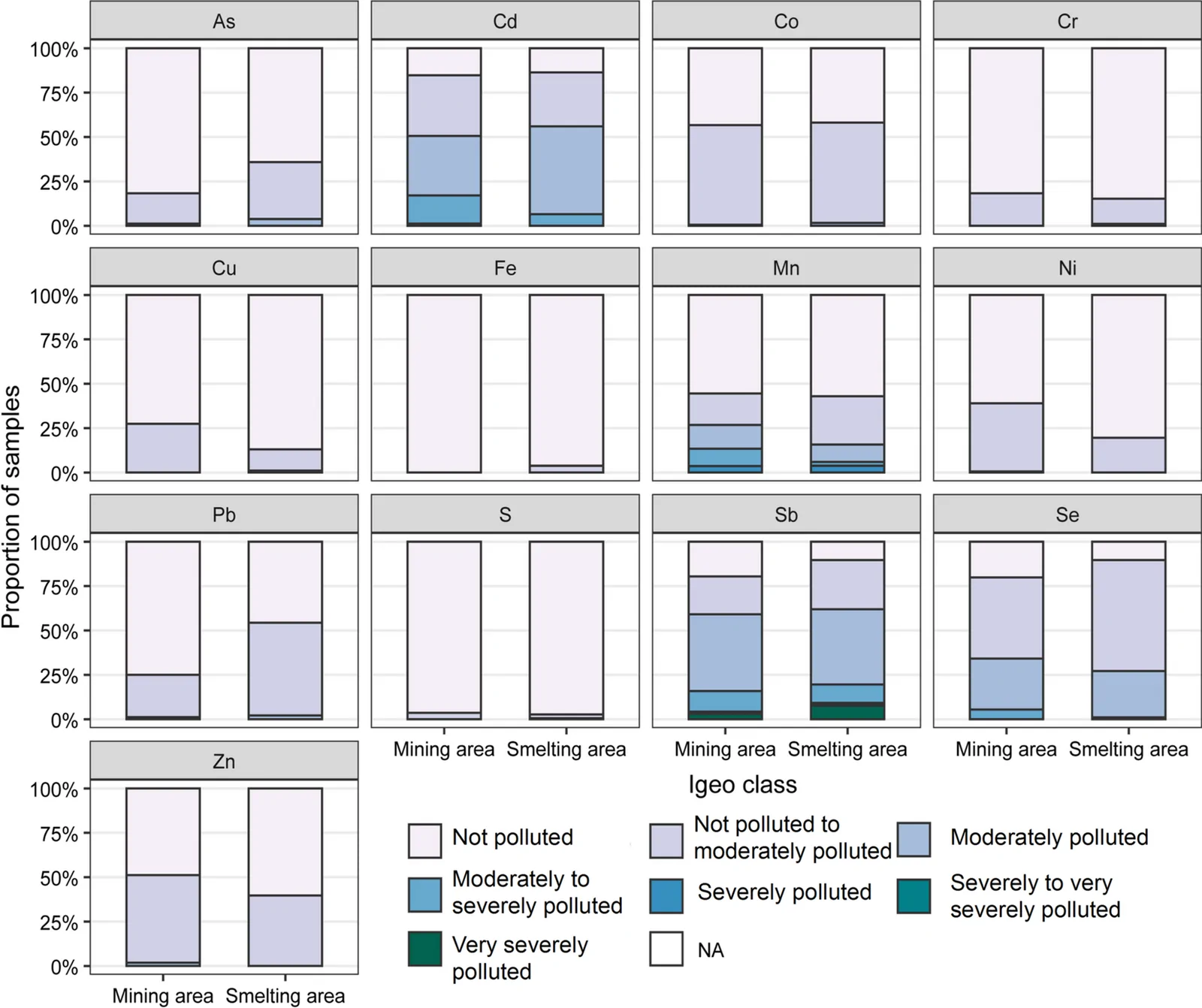
Distribution of *Igeo* classes across soil samples in the mining and smelting areas.

Across metals, *Igeo* in both mining and smelting areas was dominated by the classes of not polluted and not polluted to moderately polluted, but several metals showed clear enrichment signals (Fig. 3). In the mining area, most elements remained in low-level pollution. 100%, 96.3%, 81.7%, 81.7%, 72.6% and 61.0% of soil samples were not polluted by Fe, S, As, Cr, Cu and Ni, respectively. While 48.8% of soil samples were not polluted, 49.4% were at the class of not polluted to moderately polluted for Zn. Notable exceptions were Cd and Sb: Cd had 33.5% moderately polluted and 17.1% moderately to severely polluted or above, while Sb reached 43.3% moderately polluted and 15.9% moderately to severely polluted or above; Mn showed intermediate risk with 13.4% moderately polluted and 13.4% moderately to severely polluted or above.

In the smelting area, most metals were similarly low-level pollution compared with the ming area. As, Cr, Cu, Ni, Zn, Fe and S did not contaminate 64.1%, 84.8%, 87.0%, 80.4%, 60.3%, 96.2% and 97.3% of soil samples, but Pb and Sb stood out for pollution. Pb shifted toward broad enrichment (45.7% in the not polluted, 52.2% in the not polluted to moderately polluted, 2.2% in the moderately polluted). Sb showed the highest severity (42.4% in the moderately polluted and 19.6% in the moderately to severely polluted or above, including 7.6% in the extremely polluted).

Se and Co in both areas clustered in the levels of moderately polluted and not polluted to moderately polluted. For Se, 28.7% and 45.7 of soil samples were at the level of moderately polluted and not polluted to moderately polluted in the ming area, whereas 26.1% and 62.5% were in the smelting area. For Co, soil samples were almost all at the levels of not polluted and not polluted to moderately polluted, but less than 1.6% of soil samples were moderately polluted by Co in both areas. Comparatively, smelting exhibits a broader and more severe tail for Sb (19.6% in the moderately to severely polluted or above, with 7.6% in the extremely polluted), and a site-wide shift for Pb (only 45.7% not polluted), whereas mining shows higher pollution (moderately to severely polluted or above) fractions for Cd (17.1%) and Mn (13.4%).

### CR contributions

CR contributions from different pathways across various soil depths in the mining and smelting areas showed consistent patterns (Fig. S2a). In the mining area, ingestion was the predominant pathway contributing to CR, with contributions of nearly 100% at 0–10, 10–20, 20–30, and 30–40 cm. Dermal exposure had a minimal contribution to carcinogenic risk, and inhalation had a negligible contribution. At a depth of 0–10 cm, the contributions to CR were 99.5% for ingestion, 0.01% for dermal exposure, and 0.004% for inhalation. In the smelting area, ingestion, dermal exposure, and inhalation accounted for 96.7%, 2.4%, and 0.4% of the CR in the 0–10 cm soil layer, respectively. In deeper soil layers, ingestion was still the dominant contributor to CR but the contribution from dermal exposure showed a slight increase, particularly at a depth of 30–40 cm depth. These results show that ingestion is the dominant pathway contributing to CR in both areas, while dermal exposure and inhalation have minimal contributions across all soil depths.

### HI contributions

In the mining area, dermal exposure was the dominant contributor to the HI, with a contribution of 78.4% at a depth of 0–10 cm. This was followed by ingestion, with a contribution of at 21.5% (Fig. S2b). Inhalation had a minimal contribution (0.2%). At depths of 10–20, 20–30, and 30–40 cm, dermal exposure remained the primary contributor to the HI, and there were slight variations in the ingestion contribution. In the smelting area at a depth of 0–10 cm, dermal exposure was also the dominant contributor to the HI (79.9%), followed by ingestion (19.9%), and inhalation (0.2%). Dermal exposure remained the primary contributor across all soil depths, with a contribution of 73% at 30–40 cm. Ingestion consistently had the second largest contribution, which peaked at 27.8% at a depth of 20–30 cm. These findings show that dermal exposure is the predominant contributor to the HI in both areas, which is in contrast to ingestion being the primary pathway for the CR (“CR contributions” Section).

### Correlation analysis and PCA

**Fig. 4:**
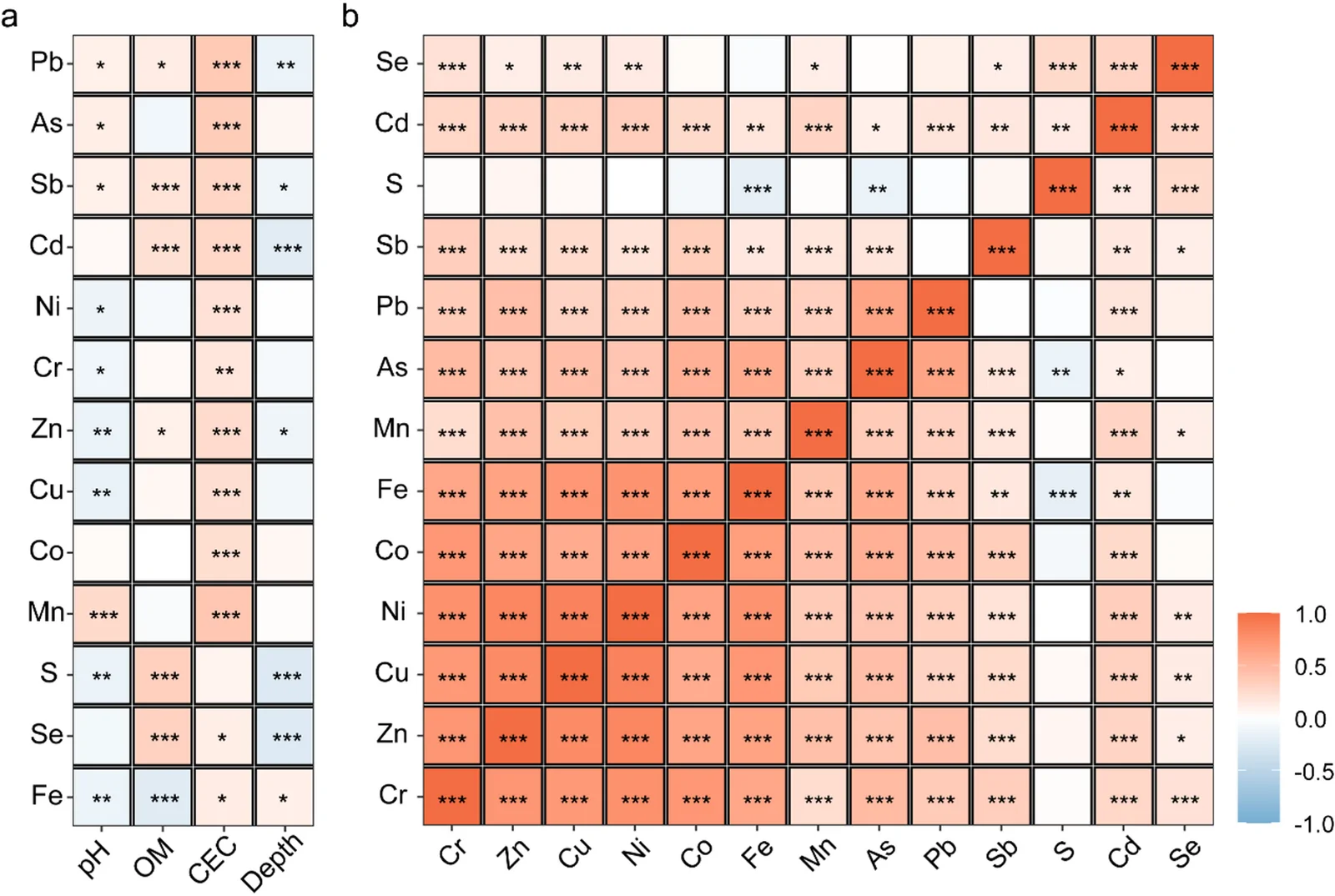
Correlations of metals with pH, soil organic matter, cation-exchange capacity, and depth (**a**). Inter-metal co-variation among transition metals and other elements (**b**).

Correlations with soil properties (Fig. 4a) indicated that the pH was positively associated with the concentration of Mn (*P* < 0.001) and negatively associated with the concentrations of Fe and Se (*P* < 0.001). Weak or minimal correlations were observed for the remaining metals. The SOM content showed strong positive correlations with the Se and Cd concentrations (*P* < 0.001) and weaker positive correlations with the Mn and Co concentrations. The CEC exhibited widespread positive correlations, notably with the Cd, Cu, Ni, Co, Mn, and Fe concentrations (*P* < 0.001). Depth was negatively correlated with the concentrations of most metals, including Mn, Ni, Cu, Zn, Cd, Pb, and Se (*P* < 0.001), and positively correlated with the concentrations of Fe and As (*P* < 0.001). The correlation between depth and S was negative significantly (*P* < 0.001). Inter-metal correlations (Fig. 4b) revealed a coherent transition metal cluster (Cr, Zn, Cu, Ni, Co, Fe, and Mn) that showed strong correlations (*P* < 0.001). The Pb concentration was positively correlated with the As concentration (*P* < 0.001) but had only a weak relationship with the Sb concentration. Collectively, the correlation matrices pointed to tight co-occurrence among the transition metals, with their concentrations modulated by the pH and CEC and depth gradients reinforcing their dependencies.

**Fig. 5:**
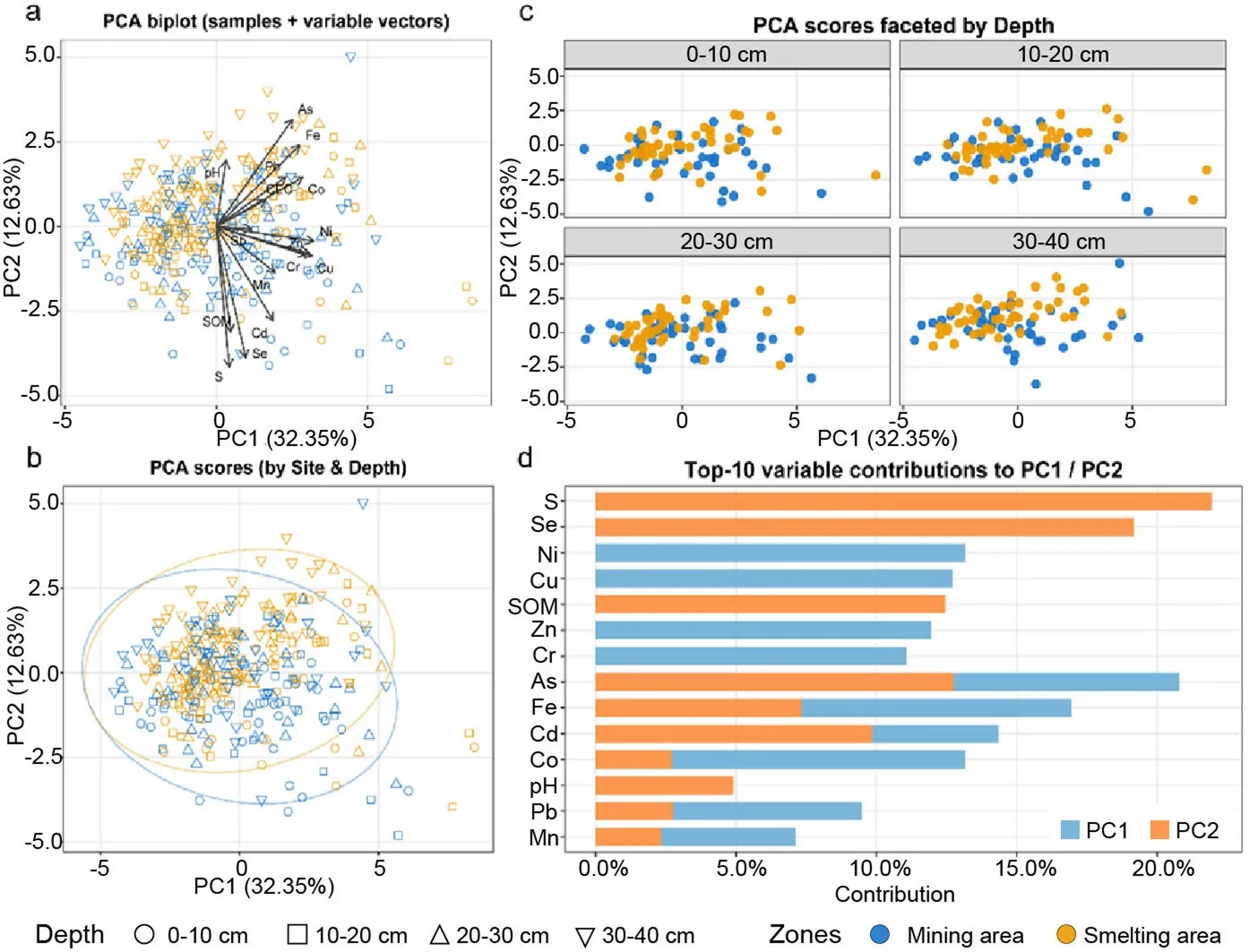
Principal component analysis (PCA) of soil chemistry in mining and smelting areas: (**a**) biplot; (**b**) sample scores by site; (**c**) sample scores by depth; (**d**) variable contributions.

PCA revealed distinct spatial patterns in the mining and smelting areas. The samples were separated along PC1 and PC2, which explained 32.35% and 12.35% of the variance, respectively (Fig. 5a). The variables in the PCA biplot indicated that elements such as Ni, Cu, Zn, and Cr were major contributors to the variability in PC1, while PC2 was influenced by elements such as S, Se, and As, which are associated with smelting areas (Fig. 5). PCA scores further illustrated that the soil depth influenced the differentiation between the areas (Fig. 5b). In the smelting area, the samples exhibited greater spread than in the mining area, especially in deeper soil layers (20–30 and 30–40 cm), which suggested there was higher variability in the contamination levels (Fig. 5b and c). Conversely, the mining area samples showed less variation across depths, which indicated that contamination was more uniform. At a depth of 0–10 cm, the PCA scores from both areas largely overlapped, but as the depth increased, the smelting area samples became more distinct from those in the mining area and metal concentrations were higher along the positive directions of both PC1 and PC2 (Fig. 5c). This was particularly apparent at depths of 20–30 and 30–40 cm. The top contributors to variability in the PCA components were Ni, Cu, Zn, and Cr for PC1, and S, Se, and As for PC2.

### Influence of soil properties on metal distributions

**Fig. 6:**
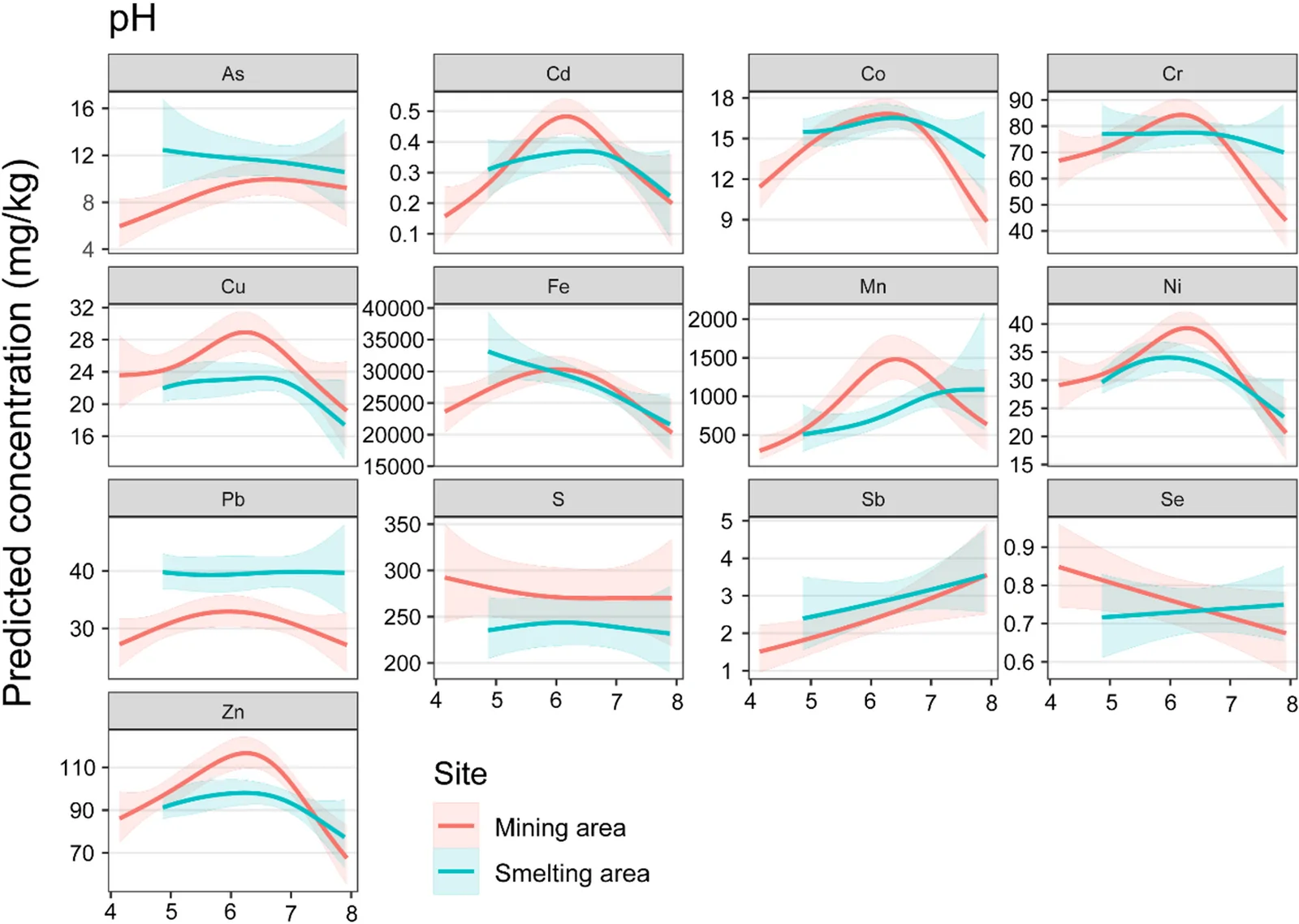
Predicted concentrations of heavy metals in soils across different pH ranges. Data show the comparative trends between the mining and smelting areas for each heavy metal, with shaded areas indicating the 95% confidence intervals.

Soil pH exerted a pronounced influence on the distribution of potentially toxic elements (PTEs) (Fig. 6). The concentrations of most transition metals, including Cd, Co, Cr, Cu, Fe, Mn, Ni, Pb, and Zn, were highest under slightly acidic to near-neutral conditions (pH 5–6.5), and then markedly decreased with increases in alkalinity. This pattern reflects the enhanced solubility and mobility of metals in acidic soils and stronger adsorption at higher pH values. By contrast, the As and Sb concentrations increased progressively with increases in the pH, suggesting they were more stable under alkaline conditions. The S concentration decreased and that of Se showed a modest increase, particularly in the smelting area.

The SOM content also shaped the PTE distributions in distinct ways (Fig. S3). Cd, Mn, and Fe displayed U-shaped responses, which indicated they were mobilized in the presence of both low and high SOM contents. The concentrations of elements with strong affinities for organic phases, such as As, Sb, and Se, increased steadily with increases in the SOM content, which highlighted the role of organic compound–mineral interactions in the retention of these metals. By contrast, the concentrations of Cu, Zn, and Ni exhibited weaker correlations with the SOM content, especially in smelting soils, which implied that their mobility was less strongly governed by organic inputs compared with the mobilities of redox-sensitive or chalcophilic elements.

The CEC was positively correlated with most PTEs (Fig. S4). The concentrations of transition metals such as Co, Cr, Cu, Fe, Mn, Ni, Pb, and Zn increased consistently with increases in the CEC, which underscores the importance of exchangeable binding sites in metal accumulation. Similarly, the Cd, Sb, and Se concentrations increased with increases in the CEC. By contrast, the S concentration increased with increases in the CEC, which showed it had different retention behavior. Notably, smelting soils showed stronger enrichment of As, Pb, and Zn relative to mining soils, which was indicative of localized contamination inputs superimposed on soil geochemical controls.

## Discussion

### Hydrogeochemical partitioning across mining–smelting landscapes

Vertical and land use contrasts in the Xiushan manganese triangle profiles reflect emission inputs, soil buffering, and element specific speciation. Smelting-derived contaminants accumulate in surface horizons, while mining-related metals follow lithogenic controls with stable vertical distributions^49^. In the smelting area, a lower pH in the upper 30 cm of the soil (6.3–6.5 vs. 6.7–6.9 in mining soils) indicated that sustained concentrations of S-containing particulate matter and metal aerosols acidified the soil horizons, promoted proton driven dissolution of Fe–Mn oxides, decreased the negative charge, and intensified competition among protons, cations, and oxyanions^50, 51^. This acidification coincided with enrichment of Pb, Zn, and As in the 0–30 cm segment and depletion of Cu and Ni at depth, and can be explained by differences in deposition flux, sorption, and redox complexation^52, 53^. Pb and Zn, which are emitted mainly as particulates or chloride/oxide complexes, partition to colloids and accumulate in the plough layer. As associates with Fe oxyhydroxides but can migrate modestly under reducing events^54, 55^. By contrast, Cu and Ni are more mobile under acidic conditions because of complexation with organic compounds and weaker sorption, which lead to depletion in subsoils relative to mining soils^56^. Concentrations of Cr and Co close to background levels and invariance of Fe, Mn, S, Sb, and Se concentrations with depth indicate that there is mineralogical control, with Fe–Mn phases acting as hosts and sinks^57^. The following correlations in the present study mirror these processes: the Mn concentration was positively correlated with pH, whereas Fe and Se were negatively correlated with pH,the Cd, Cu, Ni, Co, Mn, Fe, Pb, and Zn concentrations were broadly correlated with the CEC; the Mn, Ni, Cu, Zn, Cd, Pb, and Se concentrations were negatively correlated with depth but the Fe and As concentrations were positively correlated with depth. The PCA results highlighted these trends. PC1 (Ni, Cu, Zn, and Cr was representative of transition metals retained on Fe–Mn oxides, while PC2 (S, Se, and As was representative of smelting–redox processes driven by acidification and intermittent reduction^58, 59^. High dispersion of smelting samples at a depth of 20–40 cm reflects heterogeneous migration and microsite transformations^60, 61^. Topsoil enrichment of Pb and Zn and a lower pH implicated atmospheric deposition as the main driver, while the uniform vertical patterns and higher pH in mining soils reflected lithogenic buffering^58^. Together, the profiles (Figs. 1 and 3), correlations (Fig. 4), and ordinations (Fig. 5) support a mechanistic model where smelting intensifies acidification and Pb, Zn, and As deposition, mining sustains a Cr, Ni, Co, Fe, and Mn baseline, and the pH and CEC emerge as the main controls of speciation, sorption, and mobility.

### Risk profiling and pathway-specific exposures

The use of *P*_*i*_, *I*_geo_, PN, PERI, and health risk assessments allows for incorporation of toxicity weighting and exposure partitioning, providing a more comprehensive evaluation than the concentration alone^62^. In the present study, the *P*_*i*_ results highlighted that Sb and Cd were the dominant heavy metal pollutions, with Sb having higher *Pi* value in smelting soils and Cd higher *Pi* value in mining soils. The PERI results confirmed the importance of these elements but revealed an inversion, with Cd contributing more to ecological risk than Sb in the both areas. This inversion reflects the influence of toxicity weighting and bioavailability in the PERI results. The large toxicity coefficient and mobility of Cd in exchangeable pools increase the risk even at sub-milligram per kilogram levels, while Sb oxyanions are weakly retained at near-neutral pH values and their concentrations remain elevated under sustained deposition^63, 64^. Depth-resolved PN values revealed process contrasts. Pollution in smelting profiles was classified as severely in the range of 0–30 cm, and then decreased to low with increasing depth, which was consistent with vertical attenuation of the deposition inputs. Mining soils showed patchier distributions, with pollution classified as extremely high and relatively high in surface soils near haul roads or ore sites, which was attributed to the presence of localized hotspots on top of the lithogenic background^65, 66^. Health-risk partitioning showed ingestion dominated the CR because of the high oral potency of As and Pb, and dermal contact drove most of the noncarcinogenic risk via Ni and Sb uptake. The risk from inhalation was negligible^57^. Depth trends mirrored speciation with smelting topsoil containing As, Pb, and Zn yielding higher CR values, and deeper mining soils yielding moderate ecological risk but lower CR values as As and Pb concentrations decreased. Indices converged with multivariate fingerprints, which underscored two points. First, enrichment metrics or surface samples can incorrectly rank risks by ignoring nonlinear controls of the pH, SOM content, and CEC, such as the U-shaped SOM content responses observed for Cd and Mn or opposite pH trends for cations versus oxyanions. Second, receptor models (PCA and absolute principal component scores–multiple linear regression) improve the source attribution but still require geochemical priors to capture redox-driven partitioning, especially in paddy field soils where Fe–Mn cycling mobilizes As, Sb, and Se. Therefore, an integrated paradigm is needed that combines profile monitoring with toxicity-weighted indices, applies generalized additive models for nonlinear effects, and treats land-use redox regimes as first-order constraints. Management may be achieved as follows: mitigate Sb in smelting soils via emission control and phosphate inputs, mitigate Cd in mining soils through liming to reduce exchangeable pools, and reduce human exposure by monitoring food chains and limiting dermal dust contact.

### Governance implications and control priorities

From a management perspective, integrating vertical geochemistry^67^, source attribution, and pathway-specific risk supports a mechanism-anchored control strategy for mining–smelting–agriculture ecotones^68^. Three areas are apparent for control priorities: stabilization of topsoil redox–sorption conditions, decoupling of agriculture from metal cycling, and prioritization of controls that maximize reductions in the CR and HI rather than only lowering concentrations or* I*_geo_*/*PN scores. In smelting-impacted paddy fields, lower pH values in the upper 30 cm of the soil coincide with Pb–Zn enrichment and a positive As–pH slope. Modest liming toward near-neutral pH reduces cation exchange capacity and their oral and dermal bioavailabilities, which decreases the CR and HI values; however, excessive alkalinization risks stabilizing arsenate oxyanions. Because metal loads increase with the CEC, amendments such as humified organic materials, clays, or zeolites are useful, although low-solubility organic materials are preferable to avoid Cu/Ni mobilization. Water management strategies for reducing the duration of flooding curb Fe–Mn reductive dissolution, which has been identified as a driver of As and Sb release. Curbing of Fe–Mn reductive dissolution keeps oxyanions fixed to oxides and reduces plant uptake, although agronomic and methane trade-offs must be considered. In drylands near smelting corridors, atmospheric deposition dominates. Buffers and dust suppression limit Pb and Zn inputs, while surface organic materials reduce splash-driven contact. Phosphate immobilizes Pb as pyromorphite, which lowers oral bioavailability but risks As mobilization; iron-rich amendments can be used to mitigate this. Drylands close to mining areas show lithogenic Cr–Ni–Co concentrations near background levels but Cu/Ni are mobilized under acidic conditions. Maintaining the pH at 6.0–6.8 prevents leaching because this is the window where sorption is strong without mobilizing As. Exchangeable and Fe–Mn fractions can be monitored to detect mobilization during transient surges in acidity or wet years. Across land uses, ingestion dominates the CR, while dermal contact dominates the HI. These results support the use of complementary measures such as low As/Cd cultivars, Si and Fe amendments in paddy field soils, mulching and hardened paths to limit soil ingestion, and risk communication at PN/PERI hot spots. Because transition metals co-occur under pH and CEC control, soil-property engineering acts as a hub intervention. Monitoring design should include profile sampling to 40 cm, use enrichment indices alongside toxicity-weighted metrics, and apply nonlinear models to capture U-shaped SOM and monotonic CEC effects. A tiered policy could be established as follows: prioritization of smelting corridors for dust and acid fume control, management of paddy field systems through pH–redox balance and uptake suppression; and focused control of tailings and runoff in mining districts to reduce Cu mobility without inducing As release^69^. Collectively, the Xiushan profiles demonstrate that profile-aware, mechanism-based, and pathway-targeted governance can be used to achieve durable reductions in ecological and human-health risks.

## Conclusions

Soils in the Xiushan manganese triangle displayed pronounced vertical and site-specific contrasts shaped by mining and smelting activities. Smelting soils exhibited consistently lower pH values in the upper 30 cm of the soil and surface enrichment of As, Pb, and Zn, alongside significant depletion of Cu and Ni at depth. By contrast, mining soils maintained higher pH values and more stable metal distributions. Mn, Fe, S, Sb, Se, Cr, and Co showed minimal variation with depth, which was indicative of stronger lithogenic control. Pollution indices identified Sb and Cd as the principal pollutants, with Sb enrichment most pronounced in smelting soils and Cd dominating in mining soils. Ecological risk decomposition further highlighted Cd (43.0%) and Sb (38.1%) as the major contributors to potential risks. Risk classification revealed a relatively uniform pattern of moderate ecological risk in smelting soils but greater variability and localized hotspots in mining soils. Health-risk analysis emphasized ingestion as the dominant pathway contributing to the CR (> 95%), while dermal exposure had the largest contribution to noncarcinogenic risk (~ 73–80%). The risk from inhalation was negligible. Collectively, these findings underscore the distinct pollution signatures of mining and smelting soils and highlight the need for profile-based monitoring and exposure-oriented risk management strategies tailored to land-use conditions. Given that Sb and Cd are the principal pollutants, smelting areas should prioritize Sb emission control and phosphate amendments to immobilize Sb, whereas liming in mining areas can raise pH and thereby reduce Cd mobility.

## Supplementary Information

Below is the link to the electronic supplementary material.

Supplementary Material 1

## Data Availability

The authors declare that data supporting the findings of this study are available within the article.
