# Supplementary material for: Heavy metals in mining and agricultural ecotone: vertical distribution, ecological risks, and health implications
Source: Sci Rep. 2026 Jun 23;16:28682. doi: 10.1038/s41598-026-58692-8 (PMC13575123; doi:10.1038/s41598-026-58692-8)
Supplement: Supplementary file 1 [file 41598_2026_58692_MOESM1_ESM.docx]

**Fig. S1.** Proportional distribution of soil samples categorized by Nemerow pollution index (Mildly, Moderately, and Severely) across different soil depths in the mining and smelting areas (a). Proportional distribution of soil samples categorized by ecological risk (Low, Moderate, Relatively high, and Extremely high) across different soil depths in the mining and smelting areas (b).

**Fig. S2.** Contributions of exposure pathways to cancer risk (CR) and hazard index (HI) across soil depths in mining and smelting areas (a) Cancer risk (CR) and (b) Hazard index (HI).

**Fig. S3.** Predicted concentrations of heavy metals in soils across different SOM ranges. Data show the comparative trends between the mining and smelting areas for each heavy metal, with shaded areas indicating the 95% confidence intervals.

**Fig. S4.** Predicted concentrations of heavy metals in soils across different ECE ranges. Data show the comparative trends between the mining and smelting areas for each heavy metal, with shaded areas indicating the 95% confidence intervals.
